# Supplementary material for: Are carbon emissions trading and green financial instruments synergistic? -Comprehensive quantitative research based on content analysis
Source: PLoS One. 2024 Mar 7;19(3):e0298601. doi: 10.1371/journal.pone.0298601 (PMC10919601; doi:10.1371/journal.pone.0298601)
Supplement: S1 Data — (ZIP) [file pone.0298601.s001.zip › Data/地级市Z_F工作报告.zip!/╕╩╦α/╢¿╬≈╩╨/└┤╫╘╣1⁄2╓┌║┼í╛┬φ┐╦╩2╛▌═°í┐_2019.docx]

各位代表：

现在，我代表市人民政府，向大会报告工作，请予审议，并请各位政协委员和其他列席人员提出意见。

一、2018年工作回顾

2018年，在省委、省政府和市委的坚强领导下，市政府以习近平新时代中国特色社会主义思想为指导，深入贯彻党的十九大和十九届二中、三中全会精神，认真落实习近平总书记视察甘肃重要讲话和“八个着力”重要指示精神，坚持稳中求进工作总基调，以高质量发展为引领，以脱贫攻坚为统揽，锐意进取，狠抓落实，较好地完成了市四届人大三次会议确定的各项目标任务。

——脱贫攻坚成效明显。精准制定落实“一户一策”帮扶计划，总结推广“551”产业扶贫模式，构建以“7＋X”产业全覆盖为主体、以八个小微业态为补充的产业扶贫体系，带动2.4万贫困户发展产业脱贫。预计退出贫困村267个，减贫12.55万人，贫困发生率降至9.13%。

——经济运行企稳向好。预计完成生产总值352亿元，增长6%，高于上年1.8个百分点；规上工业增加值29亿元，增长6.2%；社会消费品零售总额132亿元，增长8.5%。一般公共预算收支分别完成24亿元和251亿元，增长5.5%和14.9%。

——投资拉动势头强劲。集中开工项目5批、397个。实施5000万元以上项目195个，较上年增加20个，其中产业类项目113个，占比较上年提高9.3个百分点。固定资产投资预计增长15%，位居全省前列。

——发展质量逐步提升。科技进步对经济增长的贡献率达到48%。规上工业主营业务收入利润率提高1.27个百分点。居民消费价格指数2.3%。单位生产总值能耗和主要污染物排放全面下降。高质量发展指标全省考核排名逐季攀升。

——民生福祉不断增进。财政用于民生支出211亿元，增长15.7%。省列民生实事全部办结。城镇新增就业2万人，登记失业率3.3%。城乡居民人均可支配收入预计达到24347元和7540元，分别增长8%和10%。城乡低保补助标准分别提高7.6%和6.3%，特困供养补助标准提高7.6%。城乡居民住院实际报销比例由56%提高到68.3%。岷县、漳县洪涝灾害灾后恢复重建有序推进，住房重建和维修任务基本完成。

我们主要抓了以下几方面工作：

（一）突出产业扶贫，脱贫攻坚扎实推进。制定《打赢脱贫攻坚战三年行动实施方案》，压茬推动工作落实。投入精准扶贫专项资金30亿元，较上年增长17%。七县区全部组建国有农业发展公司。新引进培育龙头企业25家，市级以上龙头企业达到161家。新建农民专业合作社3170个，所有贫困村实现2个以上合作社全覆盖。健全农业保险产品体系，开发8大类39个地方财政补贴险种，有效降低了农民发展产业的风险。培训贫困劳动力3.86万人，输转7.54万人。建成“扶贫车间”72家，吸纳1200名贫困群众就近就业。开发乡村公益性岗位10775个，覆盖全市所有贫困村；实施光伏扶贫项目159兆瓦，带动489个贫困村2.6万贫困户稳定增收；“五小工程”、乡村旅游、电商扶贫、“互联网＋”社会扶贫、新疆转移就业安置等扶贫措施有效落实。加快补齐“三保障”短板弱项，实施农村饮水安全巩固提升工程10个，41.7万人安全饮水保障水平进一步提高；建成自然村硬化路1134公里，整治农村公路“畅返不畅”637公里；改造农村C级危房11347户；实施易地扶贫搬迁7800户3.4万人。落实各类助学资金5.1亿元，义务教育阶段贫困家庭学生实现“零辍学”。家庭医生签约服务覆盖所有建档立卡贫困户，贫困人口大病集中救治率99.2%，重病兜底保障救助率97%。深化东西部扶贫协作和中央单位定点扶贫，福州市落实帮扶资金3.18亿元，实施巨菌草种植、中蜂养殖等扶贫项目219个，旷世科技等17家福州企业在定西投资兴业；7家中央单位落实帮扶资金8100万元、协调引进项目资金36亿元。

（二）奋力爬坡过坎，经济运行逆势回升。集中精力抓投资、稳工业、促消费，推动经济平稳健康发展。开展“十三五”规划中期评估，编制完成乡村振兴、综合交通和对接国际陆海贸易新通道等规划方案，谋划支撑项目2196个。实施中央预算内投资项目144个，完成投资17.3亿元。9个省列重大项目、34个市列重点项目完成年度建设任务。探索“一降两变一公开”融资模式，通定、定临、陇漳三条地方高速公路破难推进，胡麻岭隧道、渭河特大桥等控制性工程开工建设，完成投资20.4亿元。规范政府和社会资本合作，开工PPP项目13个，完成投资37亿元，新签约12个、总投资25.6亿元。完善招商引资工作机制和政策，组团参加兰洽会、海交会、敦煌文博会等重点节会，赴外专题招商20多次，新签约项目58个、签约资金188.7亿元，项目落地率、资金到位率分别为53%、19%。召开工业发展暨开发区建设大会，力促工业强市配套政策落实，工业经济下行趋势有效扭转。停产半停产企业较年初减少63户。实施宏腾油气柔性罐生产线等技改项目88个，建成投产60个。开展中小微企业培育行动，新增小微企业150户、规上工业企业15户。表彰“功勋企业家”“优秀企业家”，企业家活力进一步迸发。首次将经济开发区作为独立经济单元统计考核，提升基础配套水平和产业集聚功能，通渭工业集中区升级为省级经济开发区，定西经济开发区被评定为省级高新技术产业开发区。出台促进消费增长配套政策，编制完成市场建设五年规划，10个农产品批发市场、10个大型商品交易市场和4个县乡农贸市场建设加快推进。举办渭水文化旅游节、冬春旅游冰雪体验季、通渭书画艺术节、临洮马家窑文化节、陇西半程马拉松等文旅节会和赛事活动，带动批零住餐四大消费行业较快增长，消费对经济增长贡献率提高到76.5%。

（三）狠抓转型升级，产业发展态势良好。编制完成绿色生态产业发展规划和10个专项行动计划，十大生态产业破题起步。加快创建国家中医药产业发展综合试验区核心区，推进中药材种植、加工、仓储、应用、检测标准化建设，建立种子种苗基地9.4万亩、标准化种植基地122万亩，新版GMP认证企业达到74家，中药材交易量达到95万吨，甘肃中药材交易中心实现线上交易额5.1亿元。陇西药博园建设创造了新的“甘肃速度”，被省政府通报表扬。圆满承办首届中国（甘肃）中医药产业博览会，“中国药都”影响力大幅提升。推广马铃薯标准化种植272万亩，生产原原种10亿粒，建立原种扩繁基地8万亩、一级种扩繁基地70万亩，主食化加工能力达14万吨。马铃薯大会及马铃薯美食节办出了定西特色。建成裹包青贮生产线13条，生产商品牧草145万吨，肉蛋奶总产量达22.3万吨，种养加销一体化全产业链加快发展。安定区创建甘肃省草产业知名品牌示范区。第五届中国草业大会、全市首届赛牛相牛大会成功举办。中医药、马铃薯、草牧产业实现总产值184亿元、170亿元和170亿元，分别增长18%、4.3%和9.7%。编制完成现代种业发展规划，种子种业稳步发展。建成经济林果基地16.5万亩；蔬菜标准化种植面积70万亩。通渭风电基地20万千瓦风电场建设推进顺利，110千伏升压站主体工程基本完成。甘肃（岷县）国际陆港完成规划编制，金帆公铁联运港、通渭亿联商贸城等项目进展顺利。推动文化旅游体育融合发展，全域旅游发展十大行动全面实施，36个文化旅游项目加快推进，30个乡村旅游示范村启动建设。乐途网全域旅游体验馆上线运行，“渭水源头·李氏故里·当归定西”文化旅游品牌影响力日益扩大。定西被评为“2018避暑旅游城市观测点”。全市接待游客824万人次，实现旅游收入38.2亿元，分别增长25%和29%。

（四）严守发展底线，生态环境不断改善。启动大规模国土绿化行动，完成林业重点工程36.5万亩，城镇面山和生态廊道绿化13.3万亩，义务植树2061万株。渭河源区生态保护与综合治理、福州·定西生态林二期及扩建工程进展顺利。实施国家水土保持重点工程等水保项目51个，新修梯田11.5万亩，新增水土流失综合治理面积311平方公里。坚守耕地保护红线，基本农田保护面积稳定在934万亩以上。理顺引洮工程运行管理机制，推进引洮水资源高效利用，争取到生态扶贫三年用水指标1亿立方米。推行河（湖）长制，坚持放水、控水、用水、治水、保水“五水共治”，渭河、洮河、关川河、牛谷河、漳河“一河一方案”启动实施，河湖乱占、乱采、乱堆、乱建“清四乱”专项治理扎实开展。淘汰整治10蒸吨及以下燃煤小锅炉212台，建成煤供中心8个、二级配送网点431个，初步实现优质煤统一配送、集中管理。市区大气立体网格化监测体系建成运行。市区空气优良天数占比达90%。陇西、临洮污水处理厂改扩建加快推进，渭源会川污水处理工程基本建成，城镇生活污水处理率达90%。加强与中国环科院技术合作，马铃薯淀粉加工汁水还田循环利用试验成功。城市饮用水源地和地下水监测点、地表水断面水质100%达标。排查整治固体危险废物污染，开展废旧地膜回收利用，推进畜禽禁养区划定工作，实施岷县沟麻背土壤污染治理等项目，土壤环境质量不断改善。中央环保督察反馈问题年度整改任务全面完成。

（五）坚持建管并举，城乡面貌日益改观。加快城乡一体化进程，城镇化率达到36.25%，较上年提高1.92个百分点。高起点推进规划编制工作，完成市区新老城区和西川、南川、北川片区控制性详规及燃气、海绵城市等专项规划编制。建制镇控制性详规覆盖率达75%，村庄建设规划编制完成90%。实施“十大城建工程”项目269个，完成投资124亿元，分别增长54.6%、25.1%。实施棚户区改造2.47万套，基本建成1.78万套。市区坚持旧城抓改造、新城抓配套，重点推进12个行政文化商务片区项目，综合客运枢纽、凤翔园绿化景观等5个项目开工建设，定西现代科技园等7个项目前期加快推进。中华路市民公园开工建设，市体育场主体工程基本建成，市体育馆、城市展览馆正式启用。完成定临公路城区段改造，中华路南段、友谊北路、城南三路及桥梁竣工通车。重视解决城区供热、供水、供电问题，完成46个老旧小区供热管网维修改造，为2个老旧小区加装电梯20部。落实《定西市物业管理条例》，物业管理服务有所改进。各县实施城建项目198个，完成投资89.3亿元，通渭西环路桥梁工程、陇西仁寿山生态公园、渭源城区供水、临洮太石镇污水收集管线、漳县农村垃圾填埋场、岷县威尼斯水城建设等一批重点项目顺利实施。建成美丽示范村51个、环境整洁村155个。开展全域无垃圾城乡环境综合治理，铁路、公路、河道和城乡结合部“三边一部”治理成效明显。马营至马街公路定西过境段等10条普通省道、渭源至五竹等2条旅游公路建设积极推进。天定高速定西北出口完成改扩建。陇西、安定创建为全国“四好农村路”示范县。实施引洮二期配套、农村饮水安全巩固提升等重点水利项目49个，完成投资6.1亿元。

（六）创新体制机制，改革开放步伐加快。落实“三去一降一补”政策，商品住宅去库存化周期降至8.4个月，减免税费8.12亿元。“放管服”改革成效明显，市民服务中心启动运行，一体化网上政务服务平台建成，市级通用行政许可事项精简30%，市县区80%以上政务服务事项实现“最多跑一次”。推行项目审批10项配套改革措施，投资项目审批时限压缩60%以上。商事制度改革持续深化，“多证合一”“证照分离”全面实施，企业开办时间压缩至5个工作日。阳光招标采购平台建成运行。农村“三变”改革深入推进，入股各类资源资金资产12.3亿元，参与农民45.2万人，获得入股分红5098万元。农村集体产权制度改革整市推进试点进展顺利，清产核资全面完成，产权交易体系初步建立。国企和国有资产管理体制改革扎实推进，市属国有企业纳入市国资委统一监管，国有企业收入分配机制进一步健全。财税金融改革积极开展，国地税征管体制改革全面完成，预算绩效管理等重点改革启动实施。国投、城投、水投运营规范有序，定西金控组建运营。国投集团企业债券发行基础工作准备就绪。蓝天公司等82家企业在甘肃股权交易中心挂牌，凯凯农科在“新三板”挂牌。支持民营经济加快发展，新增非公经济市场主体2.4万多户。建成科技企业孵化器等“双创”平台10个，新认定高新技术企业7家，争创省级科技创新型企业16家。种业装备院士专家工作站、草牧业院士专家工作站挂牌运行。主动融入“一带一路”对接国际陆海贸易新通道，加强与四川凉山州等友好城市和中建材、华为、康美、珍宝岛等企业合作，对外开放广度深度进一步拓展。成功举办首届定商籍才大会，激发了回报家乡、回乡创业的热情。培育外贸进出口企业21家，实现进出口总额1.68亿元。

（七）立足共建共享，社会事业全面进步。开展“就业服务月”“春风行动”等就业服务活动，引导1090名高校毕业生到企业就业，大学生就业率达85%。落实“全面改薄”等项目资金7.5亿元，改扩建幼儿园264所、中小学校418所。推广“名园+”模式，破解公办幼儿园入园难题，学前教育三年毛入园率达96.53%。推行“阳光课堂”教学模式，农村小规模学校办学水平有效提升。九年义务教育巩固率达到97.69%，普通高考二本上线率40.5%、提高6.5个百分点。通渭、渭源顺利通过义务教育均衡发展国家验收。职业教育“一校一品”特色化办学步伐加快，创设中药材加工等品牌专业12个，岷县创建为国家级农村职业教育和成人教育示范县。实施市医院儿童病区等卫生项目16个，累计完成投资5.35亿元。统筹推进医改重点工作，落实药品采购“两票制”，结束以药养医历史，先诊疗后付费和“一站式”即时结报服务全面推行。成功创建全国“献血先进市”“基层中医药事业发展先进市”，渭源县被命名为“全国健康扶贫示范县”。为10.6万特困群众提供人身意外附加重大疾病保险保障。发放民政救助资金16.1亿元，困难群众基本生活得到保障。改造提升村级综合文化服务中心367个，临洮“三馆”投入使用，渭源文化综合体主体建成。组织实施“双百千万”文化惠民活动，开展文化下乡3.6万场次。秦腔《柴生芳》《渭水医魂》获甘肃省第九届敦煌文艺奖、《许铁堂》获红梅奖。实施体育项目22个，新增公共体育场地30万平方米，公共体育场馆全部实现免费或低收费开放。竞技体育捷报频传，我市培养输送的梁瑞、姚变娃、柳想绪等优秀运动员在国内外重大赛事中勇夺冠军，定西代表团在省第十四届运动会、省第十届残运会、省第九届民运会上再创佳绩。重点行业领域安全监管切实加强，安全生产形势总体稳定。实施“质量强市”战略，质量工作基础不断夯实。推进“食安定西”建设，食品药品监管力度不断加大。“七五”普法有效开展，依法治访不断加强，扫黑除恶专项斗争深入推进，社会大局和谐稳定。民兵应急力量建设、国防动员保障水平稳步提升。外事侨务、民族宗教、地方志、地震、粮食、气象、供销以及妇女儿童、青少年、老年人、残疾人、社会组织和社会慈善等事业取得新成绩。

一年来，市政府以政治建设为统领，从严从实加强自身建设。开展“两学一做”学习教育，系统学习研讨习近平新时代中国特色社会主义思想和党的十九大精神，树牢“四个意识”，坚定“四个自信”，坚决做到“两个维护”，自觉把中央大政方针和省委、省政府及市委部署落到实处。自觉接受市人大及其常委会法律监督和工作监督、市政协民主监督、社会舆论监督，办结人大代表意见建议74件、政协委员提案177件。配合做好地方立法工作，提请市人大常委会审议地方性法规2件。扎实开展“转变作风改善发展环境建设年”和“深化放管服改革突破年”活动，严格执行“十不准”规定，行政效能不断提升。严格落实“五步三法”督查制度，开设《抓落实进行时》媒体专栏，促进工作落地落实。举办新时代定西绿色发展讲堂，实施东西部扶贫协作千人培训计划，培训各级各类人才8000多人次。认真落实全面从严治党主体责任，严格执行中央八项规定及实施细则精神，政府系统廉政建设深入推进。

各位代表！过去一年，面对经济下行压力大、自然灾害频发、诸多困难和问题叠加的严峻挑战，全市经济社会发展能够取得这些成绩，实属不易。这是省委、省政府和市委坚强领导的结果，是市人大常委会、市政协和社会各界鼎力支持的结果，是全市人民同心同德、艰苦奋斗的结果。在此，我代表市人民政府，向全市各行各业的建设者，向各级人大代表、政协委员，向各人民团体、民主党派和无党派人士，向驻定人民解放军、武警官兵、消防救援队伍和公安民警，向中央和省属在定各单位，向为定西发展作出贡献的老领导、老同志，向大力支持我市脱贫攻坚的中央有关单位及福州市，致以崇高的敬意和衷心的感谢！

各位代表！一年来的工作实践，我们深切体会到，做好政府工作，必须把党的领导作为根本保证，把坚持和加强党的全面领导贯穿于政府工作全过程，把市委的各项决策部署转化为具体措施和具体项目，确保政府各项工作始终沿着正确方向前进；必须把不甘落后作为使命追求，坚定“只有想不到、没有做不到”的信念，以敢死拼命的精神和砸锅卖铁的决心，在各项工作中奋力赶超，努力缩小区域发展差距；必须把统筹兼顾作为重要方法，正确处理脱贫攻坚与其他工作的关系，处理当前稳增长与长远打基础的关系，处理发展经济与改善民生的关系，推动经济社会平稳健康发展；必须把真抓实干作为鲜明底色，发扬钉钉子精神，实之又实担好应尽之责，以实干推动发展、积蓄后劲、赢得未来；必须把过程管理作为有效举措，坚持先立规再干事，谋定而后动，事前精心设计，事中严格督导，事后总结考评，紧盯落实过程，力争最好结果；必须把攻坚破难作为应有担当，对发展中遇到的一切难题，拿出逢山开路、遇河架桥的闯劲，用市场思维和改革办法逐一破解；必须把群众满意作为检验标准，把群众满意不满意作为政府工作的“晴雨表”，用心用情用力解决好群众普遍关心的民生问题，让群众在发展中有真切获得感。

各位代表！2018年是改革开放四十周年。四十年来，在党中央、国务院的亲切关怀和省委、省政府的正确领导下，定西历届班子团结带领300万勤劳朴实的陇中儿女，借着改革开放的东风，大力弘扬“三苦”精神，坚持不懈与贫困抗争，全市贫困面由1982年的78.17%下降到9.13%。特色产业发展鼓起了农民的钱袋子，水泥路通到了群众家门口，160多万人吃上了甘甜的洮河水，广大群众住进了宽敞明亮的砖瓦房，两万平方公里的陇中大地旧貌换新颜。定西“一方水土养活不了一方人”的历史一去不复返，定西整体解决区域性贫困的梦想即将变为现实，定西比以往任何时候都更接近全面小康目标。我们对定西过去的发展成绩倍感自豪，对定西未来的发展前景充满信心!

各位代表！我们清醒地认识到，全市经济社会发展中还有诸多困难问题，政府工作依然存在一些薄弱环节。一是脱贫攻坚任务艰巨。全市还有贫困人口23.97万人，其中70%居住在深度贫困区域，越往后脱贫难度越大。二是发展质量依然不高。传统产业转型升级步伐较慢，生态产业处在起步阶段，工业经济基础薄弱，现代服务业发展滞后，新产业、新动能、新模式发展缓慢。三是基础设施欠账较大。新型城镇化水平较低，城镇承载服务带动功能不强。交通、水利、能源等领域重大项目推进难度大。环境污染和破坏的隐患仍然较多，生态环境保护治理不容懈怠。四是公共服务保障能力较弱。群众就业、上学、医疗、养老、城市管理、安全生产、社会治安、食品药品安全和公共文化产品供给等方面还存在不少短板，与广大群众的期盼还有较大差距。五是体制机制不够完善。对外开放程度低，科技、人才、资金制约明显，群众和企业对营商环境的满意度还不高。六是作风顽疾依然存在。政府有的部门和干部服务意识不强，“官本位”思想严重，思维观念封闭保守，开拓创新魄力不足，破解难题办法不多，工作落不实、不愿干工作的现象仍然存在。对此，我们将坚持问题导向，认真加以解决，努力把政府工作做得更好，决不辜负全市人民的期望和重托。

二、2019年重点任务

2019年是新中国成立70周年，是决胜全面建成小康社会、实现第一个百年奋斗目标的关键之年。我国经济运行保持在合理区间，整体向好的基本面没有改变。全省经济运行总体平稳，经济结构调整步伐加快，发展质量效益稳步提升。全市主要经济指标止滑回升，有效投资带动效应持续释放，重点工作推进有章可循、有路可走、有手可抓。只要我们抢抓国家和省上政策利好机遇，坚定信心，迎难而上，就一定能够开创定西发展新局面。

政府工作的总体要求是：以习近平新时代中国特色社会主义思想为指导，深入贯彻党的十九大和十九届二中、三中全会精神，认真落实省第十三次党代会、市第四次党代会安排部署，坚持稳中求进工作总基调，以高质量发展为引领，以供给侧结构性改革为主线，落实“巩固、增强、提升、畅通”八字方针，牢牢把握脱贫攻坚和生态环保两项基础性底线性任务，集中突破项目建设、生态产业、工业发展、对外开放，着力加强城乡建设、科技人才、民生保障、社会治理，全力打造“中国药都”“中国薯都”“中国西部草都”和全国特色种子种业基地，努力推动绿色发展崛起，不断提高人民群众获得感、幸福感、安全感，为决胜脱贫攻坚、全面建成小康社会打下决定性基础。

经济社会发展预期目标是：按照高质量发展要求，首先确保完成两项基础性底线性任务和约束性指标，实现17.78万贫困人口脱贫，贫困发生率下降到2.36%以下，648个贫困村退出，安定、陇西、临洮、漳县、渭源5县区整体脱贫摘帽。单位生产总值能耗和主要污染物排放控制在省下达调控目标之内。在此前提下，按照稳中有进要求，生产总值增长6%，固定资产投资增长12%，规上工业增加值增长5.5%，社会消费品零售总额增长8%，一般公共预算收入增长5%，城乡居民人均可支配收入分别增长8%、10%。城镇登记失业率控制在4%以内。居民消费价格指数控制在3%以内。

重点抓好以下九方面的工作：

（一）紧紧扭住脱贫摘帽目标，推动脱贫攻坚取得决定性进展。脱贫攻坚已到攻坚拔寨、决战决胜的冲刺阶段。今年这仗打不到半山腰，明年红旗就插不到山顶上。认真落实习近平总书记“聚焦再聚焦、精准再精准”指示要求，强化市级领导统筹包抓、专责小组指导督导、县乡具体落实责任，实行“一户一策”精准化研判、具体化落实、动态化管理，推动各项政策措施精准落地。落实“551”产业扶贫模式，坚持自建外引相结合，引进培育龙头企业20户，每县区至少引进大中型龙头企业1户。实施合作社能力提升工程，培训合作社带头人1000名，规范提升合作社300个以上，创建省级示范社30个以上。统筹推进政策性农业保险增品、扩面、提标、降费，开展种养产业综合保险，有效防范自然风险和市场风险。扶持发展八个小微业态，带动脱贫4万人以上。巩固村集体经济“空壳村”消除成果，持续增加贫困村集体经济收入。加快补齐“三保障”短板，完成易地扶贫搬迁7800户3.4万人；全面消除7488户农村存量危房；完成农村饮水安全巩固提升工程三年建设任务，提高9.7万人安全供水保障水平；整治“畅返不畅”农村公路，推进自然村通硬化路建设。争取中央预算电网基建资金6亿元，加大城乡电网建设与改造力度，提升贫困村供电保障能力。推行村级公益性设施共管共享，力争90%以上贫困村生活垃圾得到治理，农村卫生厕所普及率达到30%以上。实施教育扶贫“九大精准工程”，落实贫困家庭学生各项补助政策，巩固控辍保学成果，决不让一个孩子因贫困而失学。落实建档立卡贫困人口送医上门等政策措施，最大限度减少贫困群众医疗支出。深化对口帮扶协作，对接福州市落实财政帮扶资金2.77亿元以上，实施生态林三期工程、“阳光房”建设等项目70个以上。启动“福州·定西经贸文化交流周”活动，实现乡镇结对帮扶全覆盖和百企帮百村、百村帮百村目标，推动帮扶成效向更广领域拓展。创新中央单位定点帮扶模式，重点聚焦深度贫困村和产业扶贫，着力提升帮扶成效。坚持扶贫与扶志扶智相结合，实施好精神扶贫工程，树立一批脱贫带贫先进典型，建立生产奖补、就业补助等机制，倡导勤劳致富、脱贫光荣的良好风气，激发贫困群众内生动力。加大扶贫资金投入，加强资金使用监管，提高资金使用效益。建成运行定西扶贫惠农资金监管网，深化扶贫领域腐败和作风问题专项治理。落实巩固脱贫成果相关政策，减少和防止脱贫人口返贫。严格落实脱贫验收要求，坚决防止数字脱贫、虚假脱贫。

（二）紧紧扭住稳中有进要求，夯实经济回稳向好基础。落实好计划投资、重点前期、招商引资、PPP、基础设施补短板、市列重点“六个清单”项目。市级重点抓好5000万元以上投资项目，3月底前完成101个新建项目前期工作、6月底前全部开工，157个续建项目4月份全部复工。完善重大项目领导包抓推进机制，完成省列重大项目和47个市列重点项目年度投资任务。抢抓国家支持基础设施领域补短板政策机遇，推动陇海线定西城区段改线工程、定西民用机场、临洮军民合用机场、兰州至临洮城际铁路等重大项目前期取得实质性进展。用足用好出让国有资源资产和资产证券化筹资政策，破解重大基础设施项目融资难题。加强政府和社会资本合作全过程专业服务，加快推进项目前期工作，争取更多项目落地实施。开展“项目前期谋划攻坚年”和“十大生态产业招商攻坚年”活动，谋划投资过亿元项目200个以上，加快推进55个重点前期项目，引进落实投资过亿元产业项目30个以上，力争项目履约率达到90%以上，到位资金增长8%。

实施工业强市战略，突出传统产业绿色化改造，狠抓战略性新兴产业培育壮大。实施千万元以上工业项目67个，完成工业固定资产投资30亿元。加快建设史丹利中药材专用肥生产线节能改造等49个技改项目，新建薯峰公司农产品包装生产线等22个项目。推进特色优势产业向生物制药、中医保健康养、马铃薯主食化加工等消费型工业转型，建成佛慈红日中药饮片和配方颗粒生产线等22个项目。鼓励龙头企业跨地区、跨行业、跨所有制兼并重组，打造总部型企业。培育小微企业100户以上，新增规上工业企业10户以上、产值超亿元企业5户。履行经济开发区建设管理“双五”职能，优化规划、考核、统计等体系，强化设施配套，增强服务功能。加大园区招商引资力度，积极引进新奥能源综合能源站、空气净化设备生产等高科技项目，形成特色鲜明的创新产业集群。促进市内经济开发区与中东部地区开发区结对合作。建设定西现代科技园。渭源工业集中区升级为省级经济开发区。

落实促进消费配套政策，推动消费转型升级。健全城乡消费市场体系，提高甘肃中药材交易中心、康美西部中药城等12个大型商品交易市场运营水平。加大批零市场二次招商力度，引进品牌零售企业开设连锁门店或购物中心，提升马铃薯批发市场、亿联国际商贸城等专业市场服务能力。加强与生鲜农产品直供平台合作，完善快递和冷链物流体系，推动特色农产品实现超市直购、网络直销。开展全国供应链创新与应用试点工作，打造供应链创新应用示范城市。

（三）紧紧扭住新旧动能转换，积极培育发展生态产业。制定十大生态产业“一业一策”支持政策，建立统计监测、绩效评价、督查考核制度，强化生态产业发展推进机制。编制《特色优势产业规范提升三年行动计划》，今后三年每年投资3000万元，专项用于中医药、马铃薯、草牧、种子种业等产业规范提升。

中医中药产业，深化国家中医药产业发展综合试验区核心区建设，推动中医药产业向药医并重、生物制药、康养保健转变。以陇西县为中心，打造中药原料药生产供应保障基地。围绕GAP种植、GMP加工、GSP流通、GEP检测和GLP应用，填补产业标准空白，推进全产业链开发、全过程监测、全生命周期服务。建设当归、黄芪、党参等道地药材种子种苗基地14万亩，标准化种植率稳定在80%以上。完善中药材质量安全检测体系和追溯体系，保护道地中药材品牌。发挥市农科院科研优势，破解中药材种质退化问题。发展以中药饮片、配方颗粒、中成药、生物制药为主的精深加工业，建成投产扶正药业、天士力中天药业、神威药业等企业配方颗粒生产线，开工建设采芝林中药科技产业园。加强与广州大参林药业合作，建设总投资20亿元的甘肃道地中药材加工产业园，推动道地药材由种植到加工到检测一条龙发展。中药材加工转化能力达到38万吨，中医药加工产值达到75亿元。依托甘肃中药材交易中心，推进智能云仓、交易展厅、检验检测中心建设，推动农企联动、中药材仓前仓后和线上交易线下体验结合，打造全国中药材产业链一体化供应平台，实现税收、质量两个可管控。中药材静态仓储能力和交易量均达到100万吨以上，交易额达到200亿元。启动建设通渭温泉康养谷等3个中医药康养度假基地，加快建设康美（陇西）健康小镇。岷县创建全国当归种植产业知名品牌示范区。

循环农业产业，抓好安定、临洮国家现代农业产业园建设，扶持陇西黑家岔等田园综合体建设，争取中建材智慧农业产业园落地实施，开展安定、通渭、陇西、临洮10万亩耕地休耕试点。推动马铃薯产业品种、品质、品牌“三品”统一，农业、工业、产业、事业“四业”融合，鲜食化、主食化、工业化、市场化、产业化、国际化“六化”并进。严格执行脱毒种薯标准，加快新品种引进和专用型品种推广，推进脱毒瓶苗生产供应中心建设，生产原原种12亿粒以上，原种、一级种种植面积分别达到10万亩和90万亩。加强种薯市场监管，规范种薯销售行为。引导支持新型经营主体适度规模种植，提高机械化水平，稳定马铃薯种植面积。研发推广“土豆宴”，支持鲜食化加工、主食化产品企业发展。实施薯香园马铃薯系列产品深加工等3个技改项目，促进蓝天淀粉等企业降本增效、达产达标，推动圣大方舟建立现代企业制度，拉动上下游产业集群发展。依托国家级定西马铃薯批发市场，建成智慧化交易平台。建设马铃薯种薯科研服务中心和马铃薯小镇，建成中国薯都定西博览园。坚持以草带畜，推动牧草就地转化，促进草牧一体化发展，加快建设草牧业生态循环经济试验示范区。引进新品种、推广新技术，建设优质牧草试验繁育基地3000亩。实施草牧产业园、优质牧草种子繁育、高端草产品种植、商品草应急储备集散、牧草智能云仓等草产业项目。扶持民祥、天耀、方正等企业发展壮大。新建规模养殖场（小区）100个，引进大型企业发展规模化养殖和畜产品精深加工，延长草牧业发展链条。积极推进高原夏菜标准化种植，稳步扩大林果种植面积，鼓励开展工厂化育苗、标准化种植、品牌化营销。推广全膜双垄沟播旱作农业364万亩，推进粮改饲和玉米秸秆循环利用。

文化旅游产业，实施渭源黄香沟旅游廊道、漳县遮阳山度假中心等47个文旅体开发项目，加快建设临洮狄道乡韵、岷县年家大庄等30个乡村旅游示范村，推动李家龙宫、天井峡、平长人家等景区创A升A。组建运营定西文旅体发展集团，深化与马嵬驿、行知探索、敦煌文旅等企业合作。推进贵清山/遮阳山、渭河源等景区智慧化建设。力争全年文化产业增加值增长13%以上，旅游接待人数突破1000万人次，旅游收入增长27%以上。

通道物流产业，依托国际陆海贸易新通道建设，统筹发展综合服务型物流园区及各类专业物流园区，建设甘肃（岷县）国际陆港和安定、陇西、临洮3个物流中心，加快铭峰蔬菜8000吨农产品恒温库、通渭陇川万吨果蔬保鲜库等7个冷链物流项目建设。

先进制造产业，紧盯新材料、新工艺、新技术的引进、转化和应用，实施中建材碲化镉发电玻璃生产线等24个项目，对接引进河南龙旺钢化玻璃生产线等项目，推动制造型工业上下游联动发展。

清洁能源产业，打造定西绿色能源基地，实施通渭风电基地30万千瓦风电场项目，谋划建设地热供暖、干热岩发电、生物质发电等清洁能源项目，推动产业集群发展，培育区域性能源产业增长极。

数据信息产业，积极与阿里巴巴、华为、大唐融合通信等企业合作，启动建设定西市大数据中心、呼叫中心，打造全省重要的数据产业示范基地。依托福州大学数据研发优势和福州大数据平台优势，加快“数字定西”建设，促进互联网、大数据、人工智能与实体经济深度融合，推动大数据资源在特色产业、城市治理、生态环境、民生保障等领域广泛应用。

（四）紧紧扭住城乡统筹发展，加快补齐基础欠账短板。建立多规合一、土地收储、建管联动机制，推行规建分离、拆规分离、建拆分离、配套先行模式，提高城市规划、建设、管理和运营水平。编制《定西市中心城区道路网系统规划》等专项规划，完善城市规划导则，完成临洮、渭源“多规合一”城乡总体规划。实施“十大城建工程”项目244个、完成投资139亿元以上。建设棚户区改造项目100个、2.15万套。全面开工建设市区新城区项目，重点抓好市委党校新校址等12个行政文化商务片区项目，凤翔园生态景观工程、定西体育场建成投用，完成万达广场、智慧农贸市场、中华路市民公园、定西综合客运枢纽主体工程。实施西环路二期工程、新城区与巉口快速通道。加大城市水系建设和绿化美化力度，建成中华路南段街头绿化公园、城市面山亮化及三河交汇景观工程。启动建设西川生态创新城。各县重点推进200个补齐短板、完善功能项目，力争在路网畅通、设施配套、风貌提升等方面取得明显进展。加强城市管理，坚决整治占道经营、乱搭乱建、乱停乱放等“城市病”，坚决查处破坏公共设施等行为。加大全域无垃圾城乡环境综合治理力度，加强美丽乡村基础设施和公共服务设施建设，建成美丽示范村50个、环境整洁村150个。全面推进3条地方高速公路建设，完成投资60亿元以上。年内开工建设凤合高速岷县段。完成天定高速陇西文峰出口改扩建，积极争取实施天定高速定西出口、兰临高速临洮出口改扩建项目。推行建养一体化模式，提高普通省道、旅游公路及农村公路建设养护水平。实施引洮一期水资源高效利用、引洮二期及渠系配套等45个重点水利项目。

（五）紧紧扭住发展红线底线，加大生态环境治理保护力度。全面落实习近平生态文明思想，坚定不移实施生态立市战略，扎实推进大规模国土绿化行动，实施退耕还林、三北防护林、天然林保护、黄土高原综合治理、面山绿化提升改造工程，完成造林封育30万亩，城镇面山和生态廊道绿化6万亩，全民义务植树和农村“四旁”绿化2000万株。加强渭河源区生态保护与综合治理，争取实施渭河源林草生态扶贫项目。开展“绿盾2019”自然保护区专项督查和破坏林业生态环境问题专项整治，加强自然保护区问题整改。加大森林防火、林业有害生物防治、资源管理“两防一管”力度，确保森林资源安全。实施水土保持项目47个，治理水土流失面积310平方公里。加强工业企业污染治理，推广城乡冬季清洁供暖，实施煤改气、煤改电或洁净煤替代工程，打赢蓝天保卫战。严格落实河（湖）长制，加大“五水共治”力度，强化流域水污染综合治理，开展集中式饮用水源地环境保护专项行动，规范马铃薯淀粉加工汁水还田循环利用。整治矿产资源乱采乱挖行为，完成农用地土壤污染状况详查，开展土壤污染治理与修复技术应用试点，推进废旧地膜回收、尾菜处理利用等工作。加强重点耗能企业节能监管。持续推动中央和省级环保督察反馈问题整改。

（六）紧紧扭住创新驱动发展，强化科技人才金融支撑。实施技术创新和高新技术企业培育工程，新认定高新技术企业2家以上、省级科技创新型企业5家，全市高新技术企业达到30家以上、国家科技型中小企业入库备案60家以上。加快推进国家知识产权试点城市建设，实施知识产权倍增和知识产权优势企业培育工程，专利申请受理量和授权量增长20%以上。启动运行定西科技创新研究院和6个科技创新中心，争取设立马铃薯产业院士工作站。实施科技成果转移转化“双十”计划，建成定西科技成果转化信息共享平台，培育认定众创空间和星创天地3个。出台支持科研人员创业政策，鼓励科技人员到企业兼职从事科技成果转化活动，创办领办科技型企业，扶持小微企业和创新团队加快成长。依托种业装备、草牧业院士专家工作站等科研机构和创新平台，培养科技领军人才30名、科技创新团队50个。新选派300名科技特派员到企业、农村经济合作组织开展服务。落实优惠政策，引进急需紧缺人才，留住用好本土人才，继续实施中药产业、中医事业人才培育三年行动计划和东西部扶贫协作千人培训计划。用活用好政府与社会资本合作、债券、基金、证券、中期票据、资产证券化等融资方式，破解融资难题，把政府债务率控制在合理水平。发挥市级投融资平台功能，争取发行企业债券10亿元以上。完善融资担保体系，创新担保、反担保模式，推进40亿元特色产业发展工程贷款落地，支持民营企业、农民专业合作社发展。设立中医药产业发展基金、文旅体产业发展基金和草牧业发展基金。推进金融中心建设。开展供应链金融创新与应用试点，创新区块链、供应链金融服务，全面开展“互联网+”普惠金融服务。支持高强度紧固件、扶正药业等企业加快上市步伐。发挥农村金融综合服务室作用，打通农村金融服务“最后一公里”。

（七）紧紧扭住激发活力动力，推动改革开放深化提速。深化“放管服”改革，对标新的“甘肃速度”，加快构建只去一栋楼、只进一扇门、受理一个窗、办理一张网、群众一趟跑“五个一”政务服务模式，推动“一网一门一次”改革取得实效。加大相对集中行政许可权改革力度，推进审管分离、监审分离、管监分离，市级政务服务事项再精简30%，项目审批时间压缩到120个工作日以内。全面推行“双随机一公开”监管，创新审慎监管，推进信用信息大数据分析应用，加快建立以法治为准绳、诚信为基础、企业自律和社会共治为支撑的市场监管新格局。抓好涉农各项改革，深化农村“三变”改革，开展农村集体产权制度、新型职业农民培育等国家级试点，加快推进供销社经营服务体系恢复重建，启动供销社助推脱贫攻坚和乡村振兴岷县省级试点。深化国资国企改革，优化市属国有企业法人治理结构，改组成立国有资本投资公司，组建国有资本运营公司，推动国有企业跨领域、跨地区战略重组、做大做优做强。深化财税制度改革，强化预算约束和财政绩效管理。加强与“一带一路”沿线国家地区的经贸往来和文化交流，加大特色资源宣传推介力度，扩大多层次、全方位对外开放合作。支持巨鹏、三迪等10户骨干企业扩大出口规模，鼓励企业在“一带一路”沿线国家设立特色商品展示窗口，创建国家级出口食品农产品质量安全示范区，打造外贸自主品牌和农产品出口知名品牌，进出口额增长10%以上。落实药博会配套建设项目三年计划，实施陇西火车站改扩建等28个项目。高标准办好2019中国（甘肃）中医药产业博览会、中国·定西马铃薯大会，策划办好马家窑文化节、渭水文化旅游节、通渭书画文化艺术节、西北年货展销等节会，大力发展会展经济。

（八）紧紧扭住两个毫不动摇，支持民营经济加快发展。制定出台支持民营经济快速发展的意见，积极落实减税降费等政策措施，毫不动摇鼓励、支持、引导民营经济做大做强。实施市场准入负面清单，开展市场准入限制专项清理，坚决拆除有碍民营经济发展的各种“隐形门槛”。完善中小微企业公共服务体系，支持科技型“小巨人”企业发展，促进人才、技术、信息等要素自由流动汇集。建立企业创新激励机制，用好工业企业技术改造项目奖励政策，引导企业加快产品升级换代。通过资产证券化、特许经营等方式，支持民营资本投资建设运营城镇供水、供气、污水垃圾处理、停车场等市政设施项目，盘活存量资产。引导和支持民营企业通过发行企业债券、公司债券、非金融企业债务融资工具等进行融资。大力弘扬企业家精神，加强企业家队伍建设，组织100名企业家“走出去”学习培训，促进企业家解放思想、自我变革、健康成长。落实新型政商关系“双六条”规定和领导干部联系非公企业制度，建立民营企业发展堵点问题季交办制度，坚决依法依规处理损害企业权益的行为，让企业家专心创业、放心投资、安心经营。

（九）紧紧扭住主要矛盾变化，着力保障和改善民生。落实更加积极的就业创业支持政策，新增城镇就业1.7万人以上；加大对失业人员及就业困难人员帮扶力度，零就业家庭至少有一人稳定就业。加强农民工工资清欠工作，积极创建“无欠薪”城市。落实教育振兴三年行动计划，漳县、岷县义务教育均衡发展通过国家验收。稳步推进新一轮高考改革，促进普通高中多样化、特色化办学。深化产教融合、产学研结合、校企合作，扩大职业教育集团化办学效应，提升定西理工中专等8所职业学校标准化办学水平。研究制定支持高等教育发展意见，加快定西师专改革发展步伐，稳妥推进甘肃中医药大学定西校区、定西职业技术学院、甘农大临洮应用技术学院建设。努力营造大健康环境，推开公立医院薪酬制度改革，推行医联体、家庭医生签约、智慧医疗、医养结合新模式，加大区域医学中心、中医重点学科和薄弱学科建设，保障基本公共医疗服务有效供给。推进甘肃中医药大学附属医院定西分院和定西中医医院建设。鼓励社会资本创办医养结合机构，开展中医“治未病”康养服务。落实社保、城乡低保、特困人员救助政策和农村留守儿童、老年人关爱服务工作，健全重特大疾病保障救助机制。完善养老服务体系，全面放开养老服务市场，多形式发展养老服务业。推进殡葬改革，规范殡葬服务收费管理，倡导移风易俗和文明祭祀。落实优抚安置政策，做好退役军人服务管理工作。加快市县科技文化场馆建设，完成市博物馆文物征集和布展，定西科技文化园、临洮马家窑文化展示中心、通渭文化艺术中心、岷县“三馆”等项目完成年度建设任务。实施文艺创作“六个一”工程，支持创作具有地域特色的文艺精品。加快推进县级融媒体中心和应急广播体系建设，积极推进县级文保所和非遗中心建设。探索推行市场化、专业化运营模式，提高公共文化体育场馆利用效率。备战省第十五届运动会，积极申办省第十六届运动会。深入推进“七五”普法，创新法治宣传形式，增强全民法治意识。加强和创新社会管理，强化信访维稳和矛盾纠纷排查化解，纵深推进扫黑除恶专项斗争，严厉打击各类违法犯罪活动，维护社会大局和谐稳定。打击套取贷款、非法集资、高利贷等违法违规金融活动，打好防范化解金融风险攻坚战。严格落实安全生产责任，加强建筑工地、道路交通等重点领域和薄弱环节安全隐患排查整治，坚决遏制重特大事故发生。提升防灾减灾救灾能力，保障群众生命财产安全。落实党的民族宗教政策，依法加强宗教事务管理。完成市县级增储计划,保障区域粮食安全。加强军民融合发展、国防动员、应急力量、军兵种备战基础、基层乡镇武装部规范化建设和双拥工作，全域争创双拥模范城（县）。支持工会、共青团、妇联、科协、残联等群团组织工作。全面加强地震、气象、外事侨务、人防、老龄、地方志、红十字会、慈善等工作。

各位代表！以上九个方面，既立足当前又着眼长远，是为打赢脱贫攻坚战、全面建成小康社会、有效衔接乡村振兴战略打下决定性基础的重点工作，是顺应城乡一体化发展趋势、满足人民群众日益增长的美好生活需要的基础性任务，也是全面履行政府五项职能、协调推进“四大硬支撑”和“三大软实力”的具体安排。我们将以脱贫攻坚为统揽，抓主抓重，统筹推进，奋力开创定西发展新局面。

2019年，市政府在全面完成省列民生实事的同时，将为民办好十件实事：1.继续开发乡村振兴公益性岗位。2.实施农村劳动力培训转移就业工程，确保有劳动能力的贫困户至少1人稳定就业。3.引导扶持高校毕业生到企业、农民专业合作社、村金融服务室服务。4.改造城市老旧小区，提高供热、供水、供电和物业管理服务水平。5.加快推进天然气管道建设和入户工程。6.建设定西公交总站，优化市区公交线路，合理布局公交站点，提高公交智能化水平。7.加快推进城区停车场建设，增加车位设置。8.各县城区新开发一条高品位商业步行街，打造一个高品质夜市；改造提升30个重点小城镇综合性消费市场。9.新建城区幼儿园14所，城镇义务教育阶段“大班额”控制在3%以下；优化市区新城区教育布局。10.实施农村边远地区中小学温暖工程。

三、守正创新，全面加强政府自身建设

新时代催生新使命，新使命提出新要求。我们将深入学习贯彻习近平新时代中国特色社会主义思想，树牢“四个意识”，坚定“四个自信”，坚决做到“两个维护”，认真落实习近平总书记视察甘肃重要讲话和“八个着力”重要指示精神，坚持守正创业、守正创造、守正创新。我们将以机构改革为契机，加快构建职责明确、依法行政的政府治理体系，全面提高政府行政效能。我们将把抓落实作为政府工作生命线，以作风建设为重点，全面加强政府自身建设，努力建设人民满意政府。

（一）必须“学”字为本。增强“能力不足”的恐慌感和“本领不够”的危机感，提高学习自觉性，开展好新时代定西绿色发展讲堂，系统学习互联网金融、科技创新、共享经济、数字经济、智能制造、大健康产业等新知识、新技术，坚持学思践悟、学以致用，不断提高政府系统干部驾驭工作的本领和适应时代发展的能力。

（二）必须“干”字当头。干部干部，干是当头的，既要想干愿干积极干，又要能干会干善于干。必须以不甘落后的精神干、没有不能的胆子干、不怕吃亏的心态干、高人一筹的能力干，始终保持昂扬向上的精气神，不找做不到的理由，多想干得成的办法，咬定青山不放松，确保干得成、干得快、干得好。

（三）必须“严”字铭心。认真落实全面从严治党责任，加强重点领域和关键环节廉政风险防控，把从严要求贯穿政府工作各领域、全过程。严格执行中央八项规定及实施细则精神，严守“十不准”规定。自觉接受市人大及其常委会法律监督、工作监督和市政协民主监督、社会舆论监督，高质量办理人大代表意见建议和政协委员提案。

（四）必须“实”字打底。围绕政府履职管理，建立健全责任指标体系、重点工作体系、督查考核体系、结果运用体系和组织保障体系，做到个个部门有任务、人人肩上扛担子、天天都能有事做、月月指标有提升、年年位次往前移。教育引导政府系统干部树牢实效意识，重实干、务实功、求实效，做到凡事有交代、事事有回音、件件有着落。严格执行省市精简会议、督查活动有关要求和“无会周”制度，腾出更多时间和精力抓落实。

（五）必须“细”字为常。细化分解政府工作任务，能用指标量化的用指标量化，不能指标量化的用工作标准量化，把加快发展的任务变成有数的、有形的、有内容的项目，变成可操作、可检查、可考核的事项。全面推行过程管理，事前精心制定方案，事中加强管理，事后总结评估，抓实抓细每个环节，力争实现最好结果。

（六）必须“快”字为先。置身千帆竞发、百舸争流的新时代，定西的发展不进则退，慢进也是退。既不能求稳怕乱、被动应付，也不能瞻前顾后、裹足不前，更不能怕担风险、推诿扯皮，决不能死讲程序、延误时机。必须发扬“马上就办、真抓实干”的作风和“不待扬鞭自奋蹄”的精神，努力实现后发赶超、弯道超车。

（七）必须“紧”字相随。把跟踪问效贯穿抓落实全过程，每季度召开高质量发展分析调度会，及时研究解决经济社会发展中的困难问题。改进督查考核方式，变半年、年终督查考核为季度督查考核，年底汇总奖惩。坚持正向激励、反向问责相结合，以激励催生动力、以问责划明底线，营造良好的干事创业环境。

（八）必须“高”字为标。开展“对标福州、双招双引”活动，各项工作全面向福州看齐，把高标准要求植根于心，让奋勇争先成为常态，让争创一流成为自觉。深化法治政府建设，注重发挥法律顾问和法律专家咨询论证、审核把关作用，确保政府决策依法、民主、科学、高效。

各位代表！使命在肩，定当奋力担当；蓝图绘就，更需百倍努力。让我们更加紧密地团结在以习近平同志为核心的党中央周围，在习近平新时代中国特色社会主义思想指引下，在省委、省政府和市委的坚强领导下，凝心聚力, 真抓实干，为打赢脱贫攻坚战、决胜全面建成小康社会、加快建设幸福美好新定西而努力奋斗！
